# Different Visual Weighting due to Fast or Slow Vestibular Deafferentation: Before and after Schwannoma Surgery

**DOI:** 10.1155/2019/4826238

**Published:** 2019-02-18

**Authors:** Fredrik Tjernström, Per-Anders Fransson, Babar Kahlon, Mikael Karlberg, Sven Lindberg, Peter Siesjö, Måns Magnusson

**Affiliations:** ^1^Department of Otorhinolaryngology Head and Neck Surgery, Clinical Sciences, Skåne University Hospital, S-221 85 Lund, Sweden; ^2^Department of Neurosurgery, Clinical Sciences, Skåne University Hospital, S-221 85 Lund, Sweden

## Abstract

**Background:**

Feedback postural control depends upon information from somatosensation, vision, and the vestibular system that are weighted depending on their relative importance within the central nervous system. Following loss of any sensory component, the weighting changes, e.g., when suffering a vestibular loss, the most common notion is that patients become more dependent on visual cues for maintaining postural control. Dizziness and disequilibrium are common after surgery in schwannoma patients, which could be due to interpretation of the remaining sensory systems involved in feedback-dependent postural control and spatial orientation.

**Objective:**

To compare visual dependency in spatial orientation and postural control in patients suffering from unilateral vestibular loss within different time frames.

**Methods:**

Patients scheduled for schwannoma surgery: group 1 (*n* = 27) with no vestibular function prior to surgery (lost through years), group 2 (*n* = 12) with remaining vestibular function at the time of surgery (fast deafferentation), and group 3 (*n* = 18) with remaining function that was lost through gentamicin installations in the middle ear (slow deafferentation). All patients performed vibratory posturography and rod and frame investigation before surgery and 6 months after surgery.

**Results:**

Postural control improved after surgery in patients that suffered a slow deafferentation (groups 1 and 3) (*p* < 0.001). Patients that suffered fast loss of remaining vestibular function (group 2) became less visual field dependent after surgery (*p* ≤ 0.035) and were less able to maintain stability compared with group 1 (*p* = 0.010) and group 3 (*p* = 0.010).

**Conclusions:**

The nature and time course of vestibular deafferentation influence the weighting of remaining sensory systems in order to maintain postural control and spatial orientation.

## 1. Introduction

Postural control is maintained by both feedback and feed-forward mechanisms [1]. Feed-forward mechanisms depend upon previous postural and sensory experience and involve the concept of “internal models,” whose output consists of preformed neuromuscular strategies activated in given situations automatically or voluntarily (anticipated movement) [2]. Feedback control depends on sensory inputs (vision, vestibular, and somatosensory) that are processed, integrated, and weighted to their relative importance and context in the CNS [3]. Following sensory loss, e.g., unilateral vestibular deafferentation (uVD), the individual weighting changes with the prevailing view that visual cues get more important and sometimes lead to an overreliance on vision (visual vertigo syndrome [4]). Visual weighting has been shown to change following vestibular nerve section [5] with the conclusion that if patients were visually dependent in their postural control before surgery, they became less dependent after surgery and vice versa. Vestibular surgery offers unique possibilities to study deafferentation physiology since an exact timing of the vestibular loss can be set. If significant vestibular function persists at the time for surgery, the patients will experience severe vertigo and nausea combined with clinical findings such as nystagmus, pathological head-impulse test, and ocular tilt reaction. With time, vestibular compensation processes restore the static vestibular impact on postural control and spatial orientation [6, 7], but patients do sometimes experience postural instability and suffer from dizziness, which is especially true after schwannoma surgery [8]. Disequilibrium and dizziness are common factors that greatly impact the quality of life in patients after schwannoma surgery.

At our hospital, we do a thorough vestibular work-up prior to surgery with the aim to determine what function remains. If significant vestibular function remains, the patients are offered our pretreatment program (“PREHAB”) [9]. PREHAB consists of a gradual vestibular deafferentation induced by intratympanic gentamicin installation and the performance of vestibular exercises before and after the installation [9]. PREHAB was originally designed in order to shorten the nausea of the operated patients and to promote a quick mobilization, consistent with what is known beneficial in vestibular rehabilitation [10]. The theory of PREHAB is that the acute compensation processes following an abrupt uVD with cerebellar clamping (inhibition) of the vestibular nuclei do not happen if a gradual uVD is induced, and the training allows for continuous compensation. PREHAB has been in practice since 2004 and has shown to improve postural stability in the long term (6 months after follow-up) [11]. Gentamicin has an affinity for vestibular hair cells [12] but can also cause sensory hearing loss, especially in the high frequency range [13]. This necessitates also a thorough presurgical hearing evaluation and careful selection if the individual patient is a candidate for hearing preservation surgery.

Postural control is most often evaluated by posturography, and it has been shown that the control system needs to be sufficiently challenged during any evaluation of sensory preference, since the involved sensory systems overlap each other in terms of motion detection and spatial orientation [14]. As for appreciation of the weighting of the individual sensory systems, various methods have been applied. The most common is by performing Romberg's test in quiet stance (unchallenged standing) [5]. If patients sway less when their eyes are open, then the patients are perceived as visually dependent. However, further evaluation of that test has shown a poor test to retest reliability, making it imperative to challenge the redundancy of the postural control system in order to accurately determine the importance of vision in maintaining posture [15]. Another method to assess visual weighting has been made with the use of rod and disk [16] and rod and frame [17] tests. Both methods are performed with seated subjects that asked to align a projected horizontal line “perfectly horizontal” while being disturbed either by a rotating disk or a tilted frame in order to give false visual spatial orientation. If the subjects are misled from the false information they are labeled as visual field dependent. The method has been proven to identify subjects that suffer from dizziness or visual vertigo after suffering from uVD [16]. Investigations have also found a correlation between visual field dependence and quiet stance Romberg ratio in patients [18] with deficits in their postural control system but not in healthy subjects [18, 19]. This argues that there might be a relationship between visual field dependence and sensory weighting in the postural control system, at least if the postural control is compromised in some way, and thus, in the need of visual cues.

The aim of the present study was to compare visual dependence both with regard to postural control evaluation and spatial orientation in patients suffering unilateral vestibular loss with different time frames.

## 2. Material

Between the years 2002 and 2011, 136 patients were subjected to vestibular schwannoma surgery at Lund University Hospital. Most patients underwent an initial assessment, in which the vestibular function was investigated with video-recorded head impulse test of all 3 canals of each ear, bithermal calorics, vestibular-evoked myogenic potential measured on the sternocleidomastoid muscle (cVEMP), subjective horizontal and vertical, rod and frame tests, posturography, and eye movement analyses. Most patients also came to a follow-up after 6 months, in which posturography, video-recorded head impulse test, eye movement analyses, and rod and frame test were performed. The criteria for the present study were performed posturography and the rod and frame test, both at the initial assessment and the postsurgical follow-up. There were no signs of central dysfunction (Bruns nystagmus, ataxia, etc.) either before or after surgery, since central nervous dysfunction could confound the results. Full records could be retrieved in 57 patients. The reason for incomplete data were CNS affection in 35 patients, 14 patients did not perform rod and frame prior to surgery as it was not a set routine, 13 declined follow-up due to them living far away, 12 patients had incomplete data due to computer failure or move of laboratory, 2 were diagnosed with malignancies and declined follow-up, 1 patient was not able to perform posturography after surgery with eyes closed (group 2 below), 1 patient did not tolerate vibration to the legs before surgery, and 1 did not perform the correct posturography test.

The final 57 patients were divided into 3 groups according to vestibular function before surgery and if they performed the PREHAB program.

Group 1 (27 final patients) “*no vestibular function*” consisted of patients with no detectable vestibular function prior to surgery, i.e., best caloric response <7.5°/s, pathological head impulse tests of the affected ear, and no cVEMP responses. 17 were females and 10 were males, age 58.2 ± 9.6 years. 19 were subjected to translabyrinthine extirpation and 8 to retrosigmoidal approach. Individual vHIT performance was assessed by an experienced neurootologist, and a pathological impulse was defined as a gain <0.6 and presence of catch-up eye saccades (either overt or covert). Tumor extrameatal size was 20.7 ± 8.4 mm.

Group 2 (12 patients) “*vestibular function*” consisted of patients with remaining vestibular function prior to surgery. These patients were not treated with gentamicin either because they were diagnosed before 2004 (i.e., before the presurgical gentamicin treatment was introduced *n* = 5) or because their hearing was completely or near to normal and hence candidates for hearing preserving surgery (*n* = 7). 3 were females and 9 were males, age 47.8 ± 10.6 years. 4 were subjected to translabyrinthine extirpation and 8 to retrosigmoidal approach. Tumor extrameatal size was 17.3 ± 8.1 mm.

Group 3 (18 patients) “*PREHAB*” consisted of patients with remaining vestibular function prior to surgery that were treated with intratympanic gentamicin installations [9]. The gentamicin used was buffered and with a concentration of 30 mg/ml. The number of gentamicin instillations needed differed between patients and ranged between 1 and 4, with a mean of 2.7. Dosage was not set but ranged from 0.5 to 1.0 ml depending on how much could be installed. Half of the patients were females and half males, age 50.7 ± 14.1 years. Half of the patients were subjected to translabyrinthine surgery and half to retrosigmoidal approach. Tumor extrameatal size was 16.8 ± 5.8 mm.

The decision of surgical approach was based upon location and size of the tumor and whether hearing preservation was attempted. Hearing preservation also excluded patients from gentamicin treatment, i.e., hearing mean threshold <30 dB nHL (500, 1000, 2000, and 4000 Hz) and speech discrimination >70%.

The study (2014/171) was approved by the local ethical board (EPN) at Lund University, Sweden, and the study was performed in accordance with the declaration of Helsinki, with patients giving their informed consent.

## 3. Method

Postural control was evaluated by perturbing stance while standing on a force platform (400 × 400 × 75 mm) equipped with six strain-gauge sensors. Forces and torques actuated by the feet were recorded with six degrees of freedom by a force platform. Data were sampled at 50 Hz by a computer equipped with a 16-bit AD converter. Postural perturbations were induced by simultaneous vibratory stimulation to the belly of the gastrocnemius muscles of both legs [20] Vibrations were applied to the muscles by two cylindrical vibrators (0.06 m long and 0.01 m in diameters), held in place with an elastic strap around each leg [11].

The vibration amplitude was 1.0 mm amplitude at a constant frequency of 85 Hz. The vibratory stimulation was executed according to a computer-controlled pseudorandom binary sequence (PRBS) schedule [21] for 205 seconds by turning on/off the vibratory stimulation. The PRBS schedule was composed of stimulation shift periods with random duration between 0.8 and 6.4 seconds (yielding an effective bandwidth of 0.1-2.5 Hz). Thus, the designated PRBS stimuli covered a broad power spectrum and the randomized stimulation reduced the opportunity to make anticipative and preemptive adjustments.

After informing about the test procedure, the subjects were instructed to stand erect but not at attention, with arms crossed over the chest and feet at an angle of about 30 degrees open to the front and the heals approximately 3 cm apart. Two tests were conducted at each trial occasion, eyes open, fixating on mark on the wall at a distance of 1.5 m, and eyes closed. The test order followed our set clinical procedure, always starting with eyes open followed by eyes closed. In order to minimize external disturbances, the test subjects listened to classical music relayed through headphones [11].

The rod and frame test was performed in a dark room, with the subjects seated with their heads immobilized with straps against a neck rest. On a wall 1.5 m in front of them, a 15 cm long and 2 mm wide dimly lit light bar (rod) was projected, which remotely by the patients could be rotated in the frontal plane. The instructions were to align the rod horizontally, either with no other visual influences or with the rod surrounded with a tilted frame (right and left tilted with 20°, self-illuminating 100 × 100 cm). The test procedure was set, starting with no frame, followed by frame tilt. Four measurements each were made of the subjective horizontal and in the frame, and the mean was calculated. The test had no time limit.

### 3.1. Data Analysis

The variance in recorded torque was calculated for quiet stance (0–30 seconds) and for the stimulation period (31-230 seconds). The data were normalized by squared mass and squared height since regression analysis showed dependence on those factors [22, 23]. Vibratory perturbations predominately cause sway in the anteroposterior plane, which was the only plane analyzed in the present study.

Postural stability while standing is commonly analyzed using force platforms and the movements of the center of pressure (CoP), i.e., the point of application of the ground reaction force. We present torque variance values from the force platform recordings because these values correspond directly with the energy used towards the support surface, and changes in recorded torque from the force platform correspond well to the actual body movements and posture changes induced by vibratory stimulus [24]. However, though mathematically processed differently (i.e., including anthropometrical normalization), the information gathered from the recording torque is identical to recording CoP [25]. Thus, higher torque variance equates higher energy exertion while standing and thus a poorer postural control [24]. The Romberg ratio was calculated according to the following formula [5, 15]:

(1) $$\begin{array}{c} \text{Romberg ratio}=\frac{\text{Eyes closed }\left(\text{EC}\right)\text{ torque variance}‐\text{eyes open }\left(\text{EO}\right)\text{ torque variance}}{\text{EC torque variance}+\text{EO torque variance}}\cdot 100. \end{array}$$

A ratio close to zero or negative indicates that the stability was similar or poorer with EO than with EC, i.e., visual information was less important for postural control. This formula considers the total amount of body sway during both visual conditions (EO and EC).

The rod and frame test values were normalized to the side of the tumor (ipsilesional) in test with no frame, and in the Frame test to which side the frame was tilted, i.e., if the frame tilt was tilted to the side of the tumor (ipsilesional) or to the opposite side (contralesional) [17].

### 3.2. Statistical Analysis

The anteroposterior torque variance values during quiet (unperturbed) stance and during balance perturbations were analyzed using repeated measures GLM ANOVA (general linear model analysis of variance) on log-transformed values [26]. The main factors and subsequent factor interactions analyzed for three different group compositions (i.e., no vestibular function vs. vestibular function; no vestibular function vs. PREHAB; vestibular function vs. PREHAB). The GLM ANOVA main factors were: “group” (e.g., no vestibular function vs. vestibular function; d.f. 1) groups; “pre/post” (before, after; d.f. 1) receiving surgery and in one group gentamicin treatment; and “vision” (eyes closed, eyes open; d.f. 1) during posturography.

The Mann-Whitney *U* (exact sig. 2-tailed) test was used for between-groups post hoc comparisons. The Wilcoxon matched-pairs signed-rank test (exact sig. 2-tailed) was used for within-subjects post hoc comparisons, i.e., analyzing the changes between before and after treatments.

In all GLM ANOVA analyses, *p* values < 0.05 were considered statistically significant. For the Mann-Whitney comparisons, the significant Bonferroni corrected level was set to *p* < 0.025. For the Wilcoxon post hoc comparisons, the significant Bonferroni corrected level was set to *p* < 0.05. Nonparametric statistical tests were used in all statistical evaluations since the Shapiro-Wilk test revealed that some of the obtained datasets were not normally distributed and normal distribution could not be obtained after log transformation.

## 4. Results

### 4.1. Repeated Measures GLM ANOVA Analysis of Stability

During quiet stance, visual cues improved the stability in all 3 groups (*p* < 0.001) (Table 1). However, patients with vestibular function prior to surgery performed significantly worse during posturography with eyes closed compared to the “no vestibular function” group (*p* = 0.017).

During vibratory perturbation, visual cues improved the stability in all patient groups (*p* < 0.001). Postural control improved in the “PREHAB” and the “no vestibular function” groups after surgery (*p* < 0.001). Moreover, the “PREHAB” group had generally better stability before and after surgery than the “no vestibular function” group (*p* = 0.002). Furthermore, both the “PREHAB” (*p* = 0.010) and “no vestibular function” (*p* = 0.010) groups had significantly better stability postsurgery than the “vestibular function” group.

### 4.2. Post Hoc Evaluation of Stability

**Figure 1:**
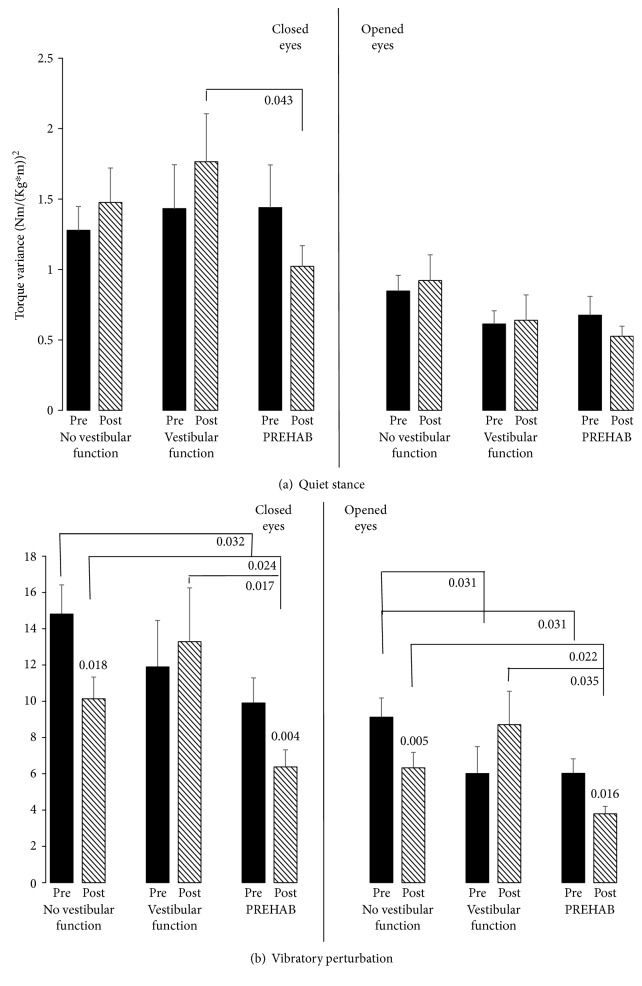
Posturography before (filled bars) and after (striped bars) schwannoma surgery in the 3 groups during (a) quiet stance and (b) perturbed stance. The values represent the mean and the error bars; standard error of mean (SEM). The Bonferroni corrected significant level for between-groups comparisons is set to *p* < 0.025 (in bold), but for consistency reasons *p* < 0.05 is also presented as trends (unbolded). The Bonferroni corrected significant level for within-subjects comparisons is set to *p* < 0.05.

None of the groups significantly changed their quiet stance stability between before and after surgery with eyes closed or eyes open (Figure 1(a)). However, a clear trend suggests poorer stability with eyes closed postsurgery in the “vestibular function” group compared with the “PREHAB” group (*p* = 0.043).

During vibratory perturbation, the patients with remaining vestibular function before surgery did not improve postural performance between before and after surgery with eyes closed and eyes open (Figure 1(b)). However, both the “no vestibular function” (*p* ≤ 0.018) and “PREHAB” (*p* ≤ 0.016) groups significantly increased performance both with eyes closed and eyes open between before and after surgery. With eyes closed, the “PREHAB” group presented trends of better stability than the “no vestibular function” group (*p* = 0.032) before surgery. After surgery, the “PREHAB” group had significantly better stability than the “no vestibular function” group (*p* = 0.024) and the “vestibular function” group (*p* = 0.017). With eyes open, trends suggest poorer stability in the “no vestibular function” group compared to the “vestibular function” (*p* = 0.031) group and the “PREHAB” (*p* = 0.031) group. After surgery, the “PREHAB” group had significantly better stability than the “no vestibular function” group (*p* = 0.022) and a trend suggests better stability than the “vestibular function” group (*p* = 0.035).

### 4.3. Repeated Measures GLM ANOVA Analysis of Romberg Ratios

The Romberg ratio during quiet stance was significantly higher in the “vestibular function” group compared with “no vestibular function” group (*p* = 0.018) (Table 2). During vibratory stimulation, there were no significant differences between the groups or before and after surgery.

### 4.4. Post Hoc Evaluation of Romberg Ratios

**Figure 2:**
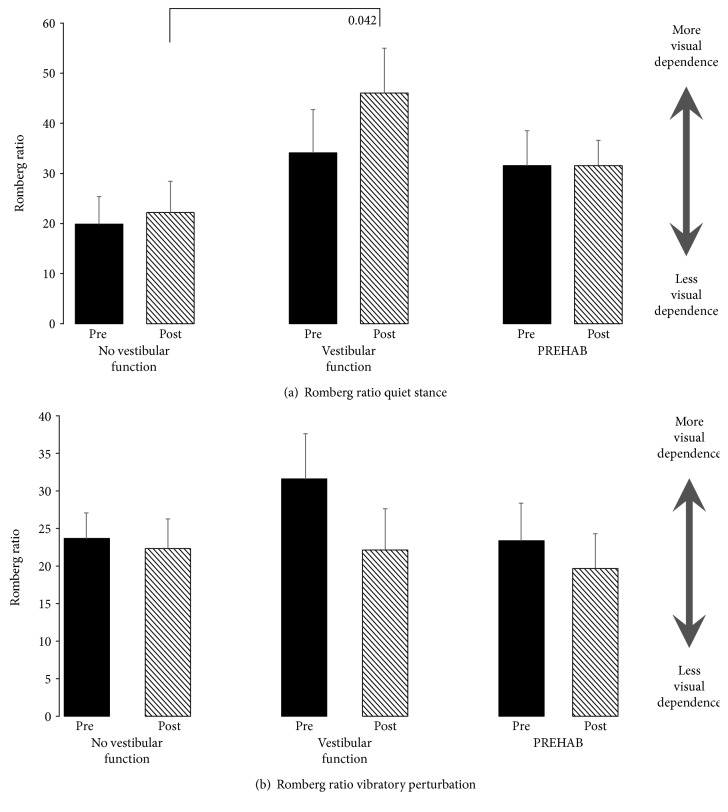
Romberg ratios during (a) quiet stance and (b) vibratory perturbation before surgery (black bars) and after surgery (striped bars). The values represent the mean and the error bars; standard error of mean (SEM). A higher value indicates a larger difference between eyes closed and eyes open conditions, and thus, more visual dependence. According to Bonferroni corrected significant level, the values were not significant, but for consistency reasons are also presented as trends (unbolded).

The Romberg ratio in quiet stance showed a trend to be higher in the “vestibular function” group compared with the “no vestibular function” group postsurgery (*p* = 0.042) (Figure 2(a)).

During vibratory stimulation, there were no significant differences between the groups or before and after surgery (Figure 2(b)). There seemed to be a tendency towards an overall reduction of the ratio, which was most evident in the “vestibular function” group.

### 4.5. Repeated Measures GLM ANOVA Analysis of Subjective Visual Orientation

The error in perceived earth horizontal increased after surgery in all GLM ANOVA group constellations (*p* ≤ 0.049) (Table 3). However, main factor interactions revealed significantly larger visuospatial errors postsurgery in the “vestibular function” group than in the “no vestibular function” group (*p* = 0.004) and significantly larger visuospatial errors postsurgery in the “PREHAB” group than in the “no vestibular function” group (*p* = 0.027). Ipsilesional frame effect was significantly lower in the “vestibular function” group compared with the “no vestibular function” group (*p* = 0.027) and the “PREHAB” group (*p* = 0.042). Contralesional frame effect was significantly lower in the “vestibular function” group compared with the “no vestibular function” group (*p* = 0.006) and the “PREHAB” group (*p* = 0.014).

### 4.6. Post Hoc Evaluation of Subjective Visual Orientation

**Figure 3:**
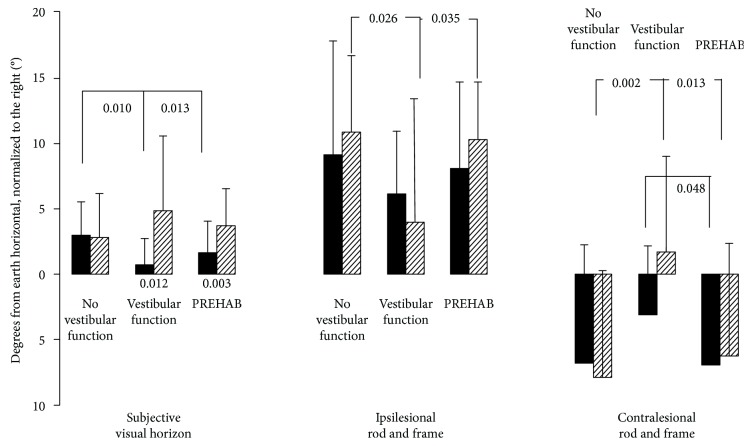
Subjective visual horizon and rod and frame tests on the three groups before (black bars) and after surgery (striped bars). The values represent the mean and the error bars; standard error of mean (SEM). The Bonferroni corrected significant level for between-groups comparisons is set to *p* < 0.025 (in bold), but for consistency reasons *p* < 0.05 is also presented as trends (unbolded). The Bonferroni corrected significant level for within-subjects comparisons is set to *p* < 0.05.

The error in perceived earth horizontal was smaller before surgery in the “vestibular function” (*p* = 0.010) and “PREHAB” (*p* = 0.013) groups than in the “no vestibular function” group (Figure 3). Moreover, trends suggest that the ipsilesional frame effect was significantly smaller after surgery in the “vestibular function” group compared with the “no vestibular function” group (*p* = 0.026) and the “PREHAB” group (*p* = 0.035). Finally, the contralesional frame effect was significantly smaller in magnitude after surgery in the “vestibular function” group compared with the “no vestibular function” group (*p* = 0.002) and the “PREHAB” group (*p* = 0.013). Furthermore, a trend suggests a smaller contralesional frame effect before surgery in the “vestibular function” group compared with the “PREHAB” group (*p* = 0.048).

The error in perceived earth horizontal was significantly smaller before surgery in the “vestibular function” (*p* = 0.012) and “PREHAB” (*p* = 0.003) groups than after surgery.

## 5. Discussion

The results suggest that postural and orientation strategies differed between the groups even though all patients suffered the same lesion (uVD). Those with “no vestibular function” lost the function probably over years due to the slow tumor growth, and the “PREHAB” group over weeks, and those with remaining vestibular function at the time of the surgery as the nerve was cut. The differences between the groups could be summarized as better postural performance with slow deafferentation and less visual field dependency with fast deafferentation. There also seemed to be a tendency with fast deafferentation for less use of visual cues to stabilize posture after surgery, which has been demonstrated earlier [27]. These findings contrast the notion that vestibular deafference lead to more visual dependence as a general paradigm.

The results suggest different central nervous compensation mechanisms to the different time courses of uVD. A quick vestibular deafference, as when the nerve is cut during surgery with remaining vestibular function or as in vestibular neuritis, leads to intense vertigo and nausea as well as the physiological features of spontaneous nystagmus and ocular tilt. These symptoms render visual cues either nauseous or confusing from the perspectives of spatial orientation and postural control, and the logical way to handle the acute situation would be to ignore visual input. On group level, this strategy seemed to persist 6 months after surgery, suggesting that it had become an integral part of vestibular compensation processes. Using available sensory cues for stabilizing posture is fundamental in feedback postural control, so it would be illogical not to use visual cues. All patients reduced their postural sway when their eyes were open, but there was a tendency that patients suffering a fast deafferentation increased their sway more with eyes closed during unchallenged posture (*p* = 0.043 vs PREHAB) and swayed more with both eyes closed and eyes open during challenging stability perturbations after surgery (*p* = 0.017 EC, *p* = 0.035 EO vs PREHAB, Figure 1). One of the reasons that the Romberg ratios did not statistically differ between the groups was that the inter- and intraindividual variation was high in all 3 groups. However, the graphic presentations of Figures 2(b) and 3 do harmonize in the sense that if challenged, the patients payed less attention to visual cues. The postural control system expects reliable sensory cues if the sensory systems could be regarded as active or functional. When standing with eyes open in darkness, healthy subjects sway more than if the eyes are closed, which illustrates the fact that the postural control system anticipate visual cues just by having the eyes open [28]. If patients suffer from nystagmus, oscillopsia, and ocular tilt in the early compensation process, then visual cues would not relay reliable information and as such could be disregarded as unimportant.

The brainstem vestibular nuclei receive afferent sensory information not only from the vestibular system but also from vision and somatosensation making the nuclei important relay stations for multisensory processing involved in sensory reweighting [29]. Also, the nuclei's connections with the cerebellum (flocculus and nodulus) are important in the central processing of sensory information [30]. Following an acute vestibular loss, the traditional view is that the cerebellum suppresses the vestibular nuclear activity bilaterally (cerebellar clamp) [31, 32], a process that gives some relief from the acute symptoms, however, also effectively shuts down the vestibular-ocular reflexes from the intact side, thus producing oscillopsia. A similar cerebellar action on vestibular compensation processes during a gradual loss (intratympanic gentamicin) has not been studied; however, since the procedure rarely produces any symptoms but rather allows patients to continue working and do their vestibular exercises, it could be assumed that the procedure does not elicit the same central nervous mechanisms. The ability to perform active head movements and not being hindered by acute oscillopsia probably enhances the cellular mechanisms involved both in the brain stem and the cerebellum during the gradual vestibular loss [33]. Recent studies have suggested a possible more direct and asymmetric impact of cerebellar activity and modification of the vestibular nuclei in rodents at least in shorter time aspects [34, 35]. It is felt however that whatever the mechanisms, a smaller vestibular error and a smaller offset to compensate per time unit should be easier to compensate for and hence reduce effort and time of the compensatory process.

The vestibular exercises before and after surgery (performed by all patients) aim at inducing motor learning at a cellular level before the vestibular loss, in line with multiple plasticity mechanisms active in cerebellar and hippocampal adaptation [36–38], as well as to familiarize the patients with the exercises to facilitate postsurgery training. Performing the exercises simultaneously as the remaining vestibular sensory information on the schwannoma side gradually diminishes due to the gentamicin treatment [39] enabled patients in the vestibular PREHAB group to continuously compensate and adapt [40]. This prepared the patients for the resultant sensory context (unilateral vestibular deafferentation) as well as to prime the postural control system to function in a new sensory context. The patients that had their vestibular function attenuated over years (slow tumor growth) with all probability also benefitted from a continuous adaptation. Even if not performing vestibular exercises, daily life activities encompass head movements of many different patterns and frequencies. Patients that are physically active before surgery are posturally more stable after surgery [41], which could be attributed to a continuous slow habituation as the function gradually declines, mirroring the gentamicin effect. In contrast, exercises performed by patients with remaining vestibular function at the time of surgery theoretically solidify the existing sensory preference which will abruptly change during surgery and could be regarded as counter productive. However, the familiarization with the exercises before surgery is an important factor for the postsurgical rehabilitation [42]. Furthermore, the nausea and vertigo in acute vestibular loss and the fact that any head movements increase symptom load restrict patients to bed causing loss of the early important mobilization for vestibular compensation processes [43]. If the deafferentation occurs gradually, then the above are not an issue. The patients continually compensate through the gradual attenuation of vestibular function and become mobilized quickly, often the day after surgery.

Separating the sensory trauma (uVD) from the surgical trauma in the PREHAB program might be beneficial for compensation processes in general. It is well known that surgery interferes with central structures such as hippocampal function [44, 45] and memory function [46, 47]. Furthermore, spontaneous nystagmus has been reported to be present on the 8th day after translabyrinthine surgery, well surpassing the time in which unimpaired compensation processes should have suppressed the physiological response [48]. It might be that compensatory plastic changes throughout the central nervous system are hindered by the combination of stress load (perioperative stress+sensory loss) and exceed the beneficial aspects of stress [49] and thus delaying compensation processes.

## 6. Conclusion

It seems that the different time courses of unilateral vestibular deafferentation affect postural competence, and these performance differences could be the consequence of different sensory weighting differences. This should be considered when designing rehabilitation programs for patients suffering from uVD. Visual dependency or not does not necessarily dictate postural performance nor dizziness, but in order to help each patient in their rehabilitation, it may be of importance to assess the individual sensory components of the postural control system and spatial orientation.

## Figures and Tables

**Table 1:** Effects of group, pre/post treatments, and vision on the stability during quiet and perturbed stance. Repeated measures GLM ANOVA analysis of how the quiet stance and perturbed stability were affected by main factors “group,” “pre/post” treatments, and “vision” alone and by the main factor interactions. The notation “<0.001” means that the *p* value is smaller than 0.001. The *F* values are presented within the squared parenthesis below the *p* values.

		Group	Pre/post	Vision	Group x pre/post	Group x vision	Pre/post x vision	Group x pre/post x vision
Quiet stance	No vestibular function vs. vestibular function	0.546 [0.4]	0.777 [0.1]	<0.001 [61.3]	0.883 [0.0]	0.017 [6.2]	0.195 [1.7]	0.388 [0.8]
	No vestibular function vs. PREHAB	0.169 [2.0]	0.523 [0.4]	<0.001 [64.2]	0.425 [0.6]	0.147 [2.2]	0.952 [0.0]	0.606 [0.3]
	Vestibular function vs. PREHAB	0.644 [0.2]	0.667 [0.2]	<0.001 [99.3]	0.368 [0.8]	0.167 [2.0]	0.405 [0.7]	0.253 [1.4]

Vibratory perturbation	No vestibular function vs. vestibular function	0.537 [0.4]	0.479 [0.5]	<0.001 [70.3]	0.010 [7.4]	0.474 [0.5]	0.181 [1.9]	0.271 [1.3]
	No vestibular function vs. PREHAB	0.002 [10.6]	<0.001 [20.2]	<0.001 [70.6]	0.903 [0.0]	0.784 [0.1]	0.470 [0.5]	0.654 [0.2]
	Vestibular function vs. PREHAB	0.100 [2.9]	0.430 [0.6]	<0.001 [50.5]	0.010 [7.7]	0.406 [0.7]	0.113 [2.7]	0.508 [0.4]

**Table 2:** Effects of group and pre/post treatments on the Romberg quotients during quiet and perturbed stance.

		Group	Pre/post	Group x pre/post
Quiet stance	No vestibular function vs. vestibular function	0.018 [6.1]	0.336 [1.0]	0.516 [0.4]
	No vestibular function vs. PREHAB	0.111 [2.6]	0.852 [0.0]	0.850 [0.0]
	Vestibular function vs. PREHAB	0.227 [1.5]	0.468 [0.5]	0.467 [0.5]

Vibratory perturbation	No vestibular function vs. vestibular function	0.486 [0.5]	0.169 [2.0]	0.299 [1.1]
	No vestibular function vs. PREHAB	0.771 [0.1]	0.461 [0.6]	0.732 [0.1]
	Vestibular function vs. PREHAB	0.420 [0.7]	0.131 [2.4]	0.502 [0.5]

**Table 3:** Effects of group and pre/post treatments on the subjective visual orientation.

		Group	Pre/post	Group x pre/post
Subjective visual horizontal	No vestibular function vs. vestibular function	0.904 [0.0]	0.006 [8.4]	0.004 [9.5]
	No vestibular function vs. PREHAB	0.703 [0.1]	0.049 [4.1]	0.027 [5.2]
	Vestibular function vs. PREHAB	0.879 [0.0]	<0.001 [18.5]	0.154 [2.1]

Ipsilesional frame tilt	No vestibular function vs. vestibular function	0.027 [5.3]	0.838 [0.0]	0.184 [1.8]
	No vestibular function vs. PREHAB	0.636 [0.2]	0.080 [3.2]	0.779 [0.1]
	Vestibular function vs. PREHAB	0.042 [4.5]	0.993 [0.0]	0.129 [2.4]

Contralesional frame tilt	No vestibular function vs. vestibular function	0.006 [8.6]	0.244 [1.4]	0.073 [3.4]
	No vestibular function vs. PREHAB	0.732 [0.1]	0.905 [0.0]	0.515 [0.4]
	Vestibular function vs. PREHAB	0.014 [6.9]	0.079 [3.3]	0.188 [1.8]

## Data Availability

The data from posturography recordings and measurement of rod and frame used to support the findings of this study are restricted by the EPN, Lund, Sweden, in order to protect patients' privacy. Data are available from the corresponding author (Fredrik Tjernström, Fredrik.Tjernstrom@med.lu.se) for researchers who meet the criteria for access to confidential data.
